# Pharmacogenetics and Predictive Testing of Drug Hypersensitivity Reactions

**DOI:** 10.3389/fphar.2016.00396

**Published:** 2016-10-21

**Authors:** Ruwen Böhm, Ingolf Cascorbi

**Affiliations:** Institute of Experimental and Clinical Pharmacology, University Hospital Schleswig-HolsteinKiel, Germany

**Keywords:** adverse drug reactions (ADRs), drug hypersensitivity reactions, drug-induced agranulocytosis (DIA), drug-induced liver injury (DILI), drug-induced severe cutaneous adverse reactions (SCARs)

## Abstract

Adverse drug reactions adverse drug reaction (ADR) occur in approximately 17% of patients. Avoiding ADR is thus mandatory from both an ethical and an economic point of view. Whereas, pharmacogenetics changes of the pharmacokinetics may contribute to the explanation of some type A reactions, strong relationships of genetic markers has also been shown for drug hypersensitivity belonging to type B reactions. We present the classifications of ADR, discuss genetic influences and focus on delayed-onset hypersensitivity reactions, i.e., drug-induced liver injury, drug-induced agranulocytosis, and severe cutaneous ADR. A guidance how to read and interpret the contingency table is provided as well as an algorithm whether and how a test for a pharmacogenetic biomarker should be conducted.

## Introduction

Apart from their intended principal therapeutic use, drugs action is always related to the risk of ADRs. ADR are an important cause of morbidity and mortality. It is estimated that 3.6% of all hospital admissions are due to an ADR and that 17% of all in-patients develop ADR, an estimated 0.5% of all ADR is lethal (Bouvy et al., 2015). The mean costs of a single ADR event in Germany has been calculated as 2,743 EUR (Meier et al., 2015). An U.S. American study reports costs from 1,439 USD to 13,462 USD (Alagoz et al., 2016). Avoiding ADR is thus mandatory from both an ethical and an economic point of view. We present the classifications of ADR, discuss genetic influences with focus on delayed-onset hypersensitivity reactions, i.e., DILI, DIA and SCAR, and present an algorithm when and how to test for relevant pharmacogenomic biomarkers.

### Taxonomy of Adverse Drug Reactions (ADRs)

**FIGURE 1:**
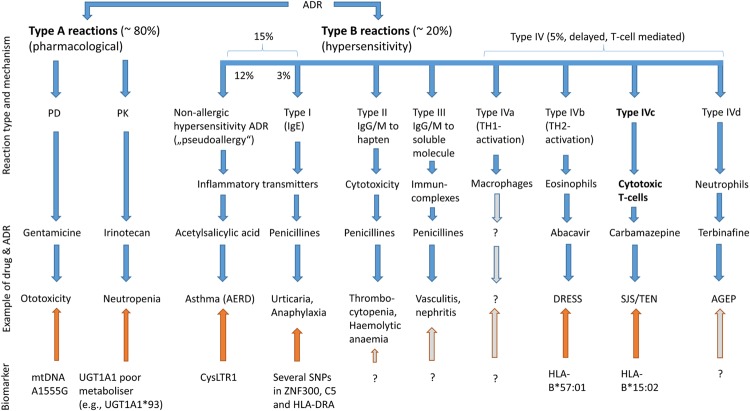
**Overview of different ADR types, examples for drugs and reactions and influencing biomarkers or patients’ conditions**.

**FIGURE 2:**
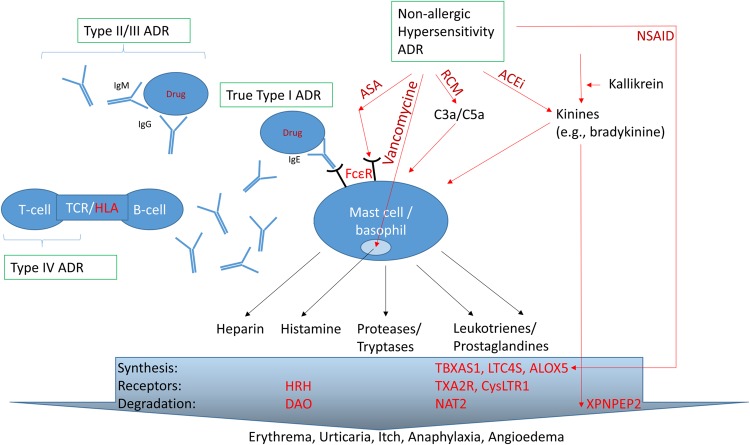
**Stimuli for degranulation of mast cells and basophils and interplay/overlap with type II-IV reactions**. Beside the canonical IgE-mediated true allergic pathway, activation of the complement system and the direct interaction with IgE-receptor can lead to degranulation. Changes in the the metabolism and signaling of various arachidonic acid-derivatives, e.g., cysteinyl leukotrienes, or in the histaminergic system, as well as changes to the kallikrein-kinine-system are believed to aggravate any reactions. Light red: proteins/genes involved in hypersensitivity with known genetic associations; Dark red: example of drugs leading to primarily non-allergic hypersensitivity ADR. ACEi, angiotensin-converting-enzyme inhibitor; ALOX5, 5′-lipoxygenase; ASA, acetylsalicylic acid (USAN: aspirin); C3a/C5a, activated components 3 and 5 of the complement system; DAO, diaminooxidase; Fc𝜀R, IgE-receptor; HLA, human leukocyte antigen; HRH, histamine receptor; LTC4S, cysteinyl leukotriene synthetase; NAT, *N*-acetyl transferase; NSAID, non-steroidal anti-inflammatory drugs; RCM, radio contrast media; TBXAS1, thromboxane synthetase; TCR, T-cell receptor; TXA2R, thromboxane receptor; XPNPEP2, aminopeptidase P.

Adverse drug reaction are divided into types A and B ADR (**Figure 1**). Type A ADR, the so-called “pharmacological ADR,” are caused (i) by a change of dosage and/or pharmacokinetics and consequently of its pharmaco- or toxicodynamic action or (ii) solely by a change in the target structure leading to different affinity of the drug to the target and/or a different agonist-directed trafficking at the (target-)receptor. In contrast, type B ADR, drug hypersensitivity ADR, are caused by allergic or non-allergic mechanisms involving the immune system and/or mediators such as histamine (**Figure 2**). Type A were estimated to account for approximately 80% of ADR occurring in clinical practice (Borda et al., 1968). However, this figure has undoubtedly changed over the last 50 years due to differences in drug prescriptions, pharmacovigilance activities and a better understanding and thus demarcation of type B ADR. 34 years later, maybe owing to these advances in medicine, type A were reported to account for 91% of all ADR (Mjorndal et al., 2002).

In the past, it was postulated that type A ADR are usually a feature of the drug property and thus predictable, while type B ADR are strongly dependent on the genetic features of the host. Pharmacogenetic polymorphisms are now known to aggravate certain type A ADR (cf. descriptions of AERD and red-man-syndrome below). Type B ADR appeared to be non-predictable and dose-independent. However, dose-dependency has been shown for some hypersensitivity reactions (Rive et al., 2013). Rising knowledge of genetic polymorphisms of the immune system have helped to predict at least some type B ADR by applying genotyping (Rive et al., 2013).

### Drug Hypersensitivity Reactions (type B ADR, Idiosyncrasy)

Depending on the mechanism of activation of the immune systems, most type B ADR (~75%) can be classified as either non-allergic hypersensitivity ADR (formerly called “pseudoallergic”), i.e., direct effect on mast cells causing histamine release, or as type I reaction according to Gell and Coombs, i.e., IgE-mediated histamine release. Type IV reactions, i.e., T-cell-mediated delayed hypersensitivity reactions, are less common (~25%). Types II and III reactions are uncommon among drug hypersensitivity reactions.

Besides these immune reactions types I to IV, a direct pharmacologic action on immune receptors (“p-i concept”) of T-cells without prior presentation of the drug via MHCs (coded by HLA) has been proposed (Posadas and Pichler, 2007). Finally, some drugs are thought to alter the functioning of the immune system, e.g., alpha-methyldopa can induce the production of drug-independent autoimmune antibodies (Pierce and Nester, 2011), and statins potentiate the shifting of T-helper 1 to T-helper 2 immune responses (Suchak et al., 2007).

### Examples, Clinical Manifestation, and Pharmacogenetics

While type A ADR are usually a feature of the drug, drug hypersensitivity reactions are strongly dependent on the genetic features of the host. However, pharmacogenetic markers have been described for both types of ADR.

#### Type A ADR

Type A ADR depend on the toxico- or pharmacodynamic action of the drug and are thus diverse. E.g., aminoglycosides are ototoxic. However, this ototoxicity can be greatly enhanced by a polymorphism in the mitochondrial DNA coding for a 12S-ribosome vital for mitochondrial protein biosynthesis resulting in complete deafness during aminoglycoside treatment (Estivill et al., 1998; Usami et al., 1998). Varying activities of drug metabolizing enzymes are the main cause of type A ADR. Classic examples for such pharmacokinetic variants is the poor-metabolizer phenotype of the drug metabolizing enzyme UDP-glucuronosyl-transferase (UGT) 1A1 which results in increased risk of neuropenia during high dose irinotecane therapy (Innocenti et al., 2009) or of cytochrome P450 2D6 causing an elevated risk of extrapyramidal symptoms while treatment with the neuroleptic haloperidol (Brockmoller et al., 2002).

#### Immediate Reactions: Type I and Non-allergic Hypersensitivity ADR

Mast cells and basophils can be stimulated to release inflammatory agents like histamine, heparin, leukotrienes, prostaglandins, cytokines, proteases, and PAF. If the stimulus derives from an IgE-antigen-complex it is considered a true type I allergic reaction. However, non-IgE-mediated responses are common and comprise approximately 77% of all reactions of this type (Demoly et al., 1999). For some drugs, both mechanisms apply (Canto et al., 2009).

After degranulation of mast cells and basophils, the resulting type I or non-allergic hypersensivitiy ADR mainly manifest in the skin as itch, urticaria, and erythema due to the pro-inflammatory substances released. Acute severe reactions, called anaphylaxia, involve the cardio-vascular system and the airways, i.e., swelling and edema of pharynx, larynx and bronchi with possible subsequent asphyxia. Anaphylaxia is seen more frequently with immediate hypersensitivity reactions than other types.

##### Non-allergic hypersensitivity ADR (“pseudoallergy”)

There are several pathways for non-IgE-mediated mast cell/basophil degranulation (**Figure 2**).

Non-steroidal anti-inflammatory drug are very commonly used drugs that are frequently involved in hypersensitivity ADR in some individuals. They are reported to be the leading cause of hypersensitivity ADR (Dona et al., 2012). ASA, USAN or NSAID-exacerbated respiratory disease (AERD or NERD, respectively) and ASA-induced urticarial (AIU) were known to affect primarily individuals with allergic rhinitis and polyposis nasi after application of an NSAID. This phenotype is also commonly referred to as Samter’s triad. The last decades shed light on various genetic markers associated with AERD/AIU, e.g., DAO (Agundez et al., 2012) and histamine-receptors (Ayuso et al., 2013). Other markers like IgE-receptors (Fc𝜀R) and alterations in synthases, receptors and degrading enzymes of CysLT and thromboxanes are nicely reviewed by (Park et al., 2013; Gomez et al., 2015). The phenotype “nasal polyposis” is associated with certain HLA genotypes (Molnar-Gabor et al., 2000).

However, predictive testing for AERD/AIU appears to date not feasible due to the multitude of possible biomarkers and their relatively weak associations: E.g., recalculating the frequency data on CysLTR1 haplotypes and AERD (Kim et al., 2006) suggests that at least approximately 300 patients need to be genetically tested to avoid one incident. Cf. below (see section ‘The Output’) for more showcase calculations and points to consider for predictive testing.

Brisk displacement of histamine from mast cells/basophils can clinically present as red-man-syndrome which is seen after rapid intravenous exposure to a variety of drugs, e.g., vancomycine, ciprofloxacine, and amphotericine B. The red-man-syndrome after application of vancomycine was linked to a missense polymorphism in the diaminooxidase (DAO) gene at c.995C > A (Myers et al., 2012). DAO is needed for efficient degradation of histamine. Defects thus cause or aggravate histamine-dependent ADR.

Mastocytosis is a pathological condition leading to large amounts of histamine being released to a variety of stimuli, including commonly used drugs like NSAIDs. While mastocytosis is poorly understood, an association with a missense variant in c-Kit gene (c.2468A > T) which encodes a tyrosine kinase receptor in stem cells is known (Nagata et al., 1995; Akin, 2006). Mutated c-Kit leads to constitutive activation of affected immune cells.

In addition to antibiotics and NSAID, other commonly used drugs or substances which can lead to non-allergic hypersensitivity ADR are radio contrast media (e.g., gadolinium, iopromid), local anesthetics (e.g., bupivacaine), opioids (e.g., morphine), curare-derivatives (e.g., rocuronium), preservatives (e.g., benzoate) and coloring agents (e.g., yellow-orange S). It appears extremely variable which of these substances actually trigger a hypersensitivity ADR in an individual susceptible patient.

Angiotensin-converting-enzyme inhibitors inhibit bradykinine degradation as off-target effect. Polymorphisms in a kinine degrading enzyme (aminopeptidase P, XPNPEP2) are thought to contribute to angioedema (Cilia La Corte et al., 2011; Mahmoudpour et al., 2013). Bradykinine is believed to worsen inflammatory responses. There are two case report of fatalities caused by allopurinol hypersensitivity possibly aggravated by concomitant captopril or enalapril, respectively (Pennell et al., 1984; Ahmad, 1995). Based on these reports, the combination of ACEi and allopurinol is considered not recommended. However, considering that this combination is extremely common and that virtually no further fatalities were reported, the mechanistic idea that ACEi will exacerbate every hypersensitivity reaction needs to be questioned.

##### True type I immediate ADR

Recently, various polymorphisms in several genes have been linked to penicillin-induced immediate hypersensitivity reactions (Gueant et al., 2015). Quite surprisingly, HLA genes appear to be involved, although HLA gene products are not prominently involved in IgE-signaling to mast cells and basophils. On the other hand, both production and specificity of IgE appear to correlate with certain HLA genes (Marsh and Bias, 1977; Young et al., 1995).

Penicillines and cephalosporins are listed in the WHO Model List of Essential Medicines and prescribed world-wide. Furthermore, after NSAID, beta-lactam antibiotics are reported to be the leading cause of hypersensitivity ADR (Dona et al., 2012) and the most frequent cause for true allergies (Blanca et al., 2009). Due to the high exposure rate and the intrinsic high risk, allergic reactions are occurring frequently.

#### Type II and Type III

Type II and type III reactions are less commonly observed. Penicillines are known to form haptens on blood cells which are subsequently targeted by IgG and IgM antibodies causing thrombocytopenia or hemolytic anemia (type II). If betalactame antibiotics a such as pencillines are bound by IgG or IgM in the bloodstream, immune complexes form and cause intra-vascular immune reactions, e.g., vasculitis, or damage the glomeruli, e.g., glomerulonephritis (type III). To our best knowledge, there is currently no data on genetic associations to such types II and III reactions.

#### Type IV

Type IV ADR may lead to symptomatic or asymptomatic internal manifestations include, among others, agranulocytosis (DIA), hepatitis (DILI), nephritis (DIRI), pneumonitis and myositis. Fever and lymphadenopathies are possible. Type IV ADR can also damage the skin SCAR, e.g., DRESSs, destruction of ~10% of the skin SJS and destruction of greater extend, the so called TEN. There is a certain overlap of DRESS, SJS, and TEN concerning the dermatological features.

Type IV ADR are strongly linked with a plethora of HLA-genes residing on chromosome 6. HLA-A, HLA-B, and HLA-C encode proteins that form a MHC I-receptor on various cell types for presentation of intracellular peptides to the immune system. HLA-DP, HLA-DM, HLA-DO, HLA-DQ, and HLA-DR are proteins for T-cell interaction, e.g., MHC II-receptors and other related proteins, consisting of alpha- and beta-chains encoded by separate genes. A single HLA-gene can be further specified, e.g., HLA-B*44:02:01:02S for a HLA-B gene of allele group 44 and allele 02. The other descriptors specify synonymous changes in the coding region, changes in the non-coding region and changes in expression. Traditional serology-based HLA typing can usually only detect the allele group of the HLA protein and not the other subtle differences below this level.

Examples of hypersensitivity-conferring HLAs and type IV ADR include abacavir + HLA-B*15:02 causing DRESS (Martin et al., 2012, 2014), carbamazepine + HLA-B*31:01 causing SJS/TEN (McCormack et al., 2011) and flucloxacillin + HLA-B*57:01 causing DILI (Daly et al., 2009). Other related genes include Transporter Antigen Processing (TAP1/2), MHC-class I related chain A/B (MICA/MICB) and hemochromatosis gene (High Fe, HFE). Data on T-cell receptors genes (TCRs) and killer cell immunoglobulin-like receptors (KIR) is scarce, but might prove relevant.

### Datasources

Several databases exist nowadays to summarize the various findings (**Table 1**).

**Table 1:** Databases of genotypes and associated ADR.

Name of database	Access
The HLA Adverse Drug Reaction Database	http://www.allelefrequencies.net/hla-adr/default.asp
LiverTox Database	http://livertox.nih.gov/
HLADR (Du et al., 2015)	http://pgx.fudan.edu.cn/hladr/

### Overlapping of Different Reaction Types

Drugs can elicit hypersensitivity ADR by several non-allergic and allergic mechanisms at once, e.g., penicillines can induce immediate and delayed ADR (Blanca et al., 2009), and vancomycin can lead to both IgE-mediated and non-allergic hypersensitivity ADR (Polk et al., 1993).

To date, the different type IV reactions are not easily separated, i.e., type IVc reactions appear to be a dominant mechanism that is also occurring during types IVa, IVb, or IVd reactions (Posadas and Pichler, 2007).

Depending on the HLA-genotype, the risk agranulocytosis mediated by the antipsychotic clozapine could be significantly increased (Goldstein et al., 2014). However, a second mechanism was proposed before hypothesizing that clozapin would be metabolized to highly reactive nitrenium ions that deplete the ATP and glutathione-content of neutrophils and would ultimately lead to neutropenia (Tesfa et al., 2009).

### Conditions Other than Genetics Influencing Type B ADR

The general state of the immune system may influence the occurrence or non-occurrence of ADR. E.g., vancomycin-related red-man-syndrome is very frequently seen (up to 90%) in non-infected, healthy patients, e.g., those receiving prophylactic treatment, but much less (3.7–47%) in those suffering from infections (Sahai et al., 1990; Sivagnanam and Deleu, 2003). Vice versa, reactions to ampicilline (notably, not amoxicilline) are much more common in EBV-infected patients (Haverkos et al., 1991; Chew and Goenka, 2016) and reactions to sulfonamides occur predominately in HIV-infected patients (Jaffe et al., 1983). The mechanism for sulfonamide-induced hypersensitivity in HIV-patients was attributed to a decrease of cellular glutathione (Rieder et al., 1995).

## Delayed-Onset Drug Hypersensitivity Reactions

### Common Features of Drug Hypersensitivity Reactions

It can be speculated whether the clinical manifestation of a drug hypersensitivity reactions is connected to specific genetic alterations. E.g., is there a common pool of polymorphisms leading to either DILI, DIA or SCAR? Existing data do unfortunately deny the existence of such a simple genetic constellation.

#### Example for One Drug – One Genotype – Several Outcomes

Allopurinol causes SJS/TEN in Han Chinese bearing HLA-B*58:01. At the same time, in the same population, it can cause DRESS.

#### Example for One Drug – Several Genotypes – One Outcome

Vice versa to the allopurinol example, nevirapine causes DRESS. It appears that the most significant HLA genotype for this reaction heavily depends on the population: HLA-B, HLA-C, and HAL-DR-loci have been associated.

#### Example for Several Drugs – One Genotype – One Outcome

Finally, phenytoin, phenobarbital, and carbamazepine can elicit SJS/TEN in HLA-B*15:02 positive patients. At least this last riddle can be explained by considering the common chemical features of the three drugs (see below for details).

### Drug-Induced Livery Injury

Flucloxacilline and amoxicilline/clavulanic acid are powerful first-line antibiotics which may cause DILI (Daly et al., 2009). Due to their wide-spread usage and inherent ability to cause DILI, it is vital to further understand the mechanism. Unfortunately for testing purposes, the numbers needed to screen are high and routine testing is (currently) not feasible. This in part due to the rareness of DILI which is estimated to occur in about 14 of 100,000 inhabitants per year (Bell and Chalasani, 2009). This comes unexpected when taking into account that the liver is a metabolically highly active organ, able to produce a variety of bioactivated and thus putatively immunogenic compounds from the parent drug. However, the liver is considered a tolerogenic environment, i.e., there are mostly no or locally restricted immune reactions in the liver (Crispe, 2003).

Amoxicilline/clavulanate has been linked to DILI in patients with HLA-DRB1*15:01 and recently with HLA-A*30:02, HLA-B*18:01 and the complex genotype HLA-DRB1*15:01-DQB1*06:02 (Stephens et al., 2013). Interestingly, the detected populations at risk also differed in age and speed of reaction onset.

### Drug-Induced Agranulocytosis

Several drugs can elicit a life-threatening destruction of blood cells. E.g., the antipsychotic clozapine is commonly (0.8% of drug users) causing neutropenia (less than 1500 neutrophil granulocytes/μl) or agranulocytosis (less than 500 granulocytes/μl). Other triggering drugs for DIA comprise thyreostatics like methimazole and the analgesic metamizole (USAN: dipyrone). Pharmacogenetic associations are known for methimazole and HLA-DRB1*08:03:2 (Tamai et al., 1996), for metamizole and various HLA genotypes (Vlahov et al., 1996) and for clozapine and a complex HLA genotype in Jewish subjects (Lieberman et al., 1990) for more than two decades.

Since clozapine appears to be the most effective antipsychotic drug available, there is a need to increase our understanding of its safe or putatively unsafe usage. The initial findings for clozapine were subsequently refined and applied to the general population (Dettling et al., 2001). Various polymorphisms leading to DIA after clozapine exposure have been identified, e.g., a complex genotype consisting of HLA-DRB5-DRB4, HLA-C-B-DRB5 (Dettling et al., 2007), HLA-DQB1 (Athanasiou et al., 2011), HLA-DQB1 (126Q) and HLA-B (158T) (Goldstein et al., 2014). The latter two studies deserve additional attention since the association was identified to be due to single amino acid changes at a defined position in the HLA gene product rather than to alleles described before. This is similar to the incidental finding that a single amino acid change in HLA-A, HLA-B, and HLA-C at position 152 might explain altered susceptibility of T-cells to drugs and lead to DILI (Stephens et al., 2013). Amino acids exchanges at this position alter the antigen binding pocket E of the MHC I receptor and possibly the interaction with T-cells. These findings suggest that a single amino acid change and not a serology-derived typing of HLA may provide a better prediction of the observed hypersensitivity reaction because of the mechanistical explanation inferred by the change in the binding pocket.

### Severe Cutaneous ADR

Next to the liver, the skin, including the mucosa, is involved in bioactivation of drugs. Additionally, since the skin is the barrier that protects our body from the environment, it is rich of immune cells. Due to this combination of a huge amount of putatively immunogenic compounds, an abundancy of immune cells and the constant pre-sensitization of the dermal immune cells due to their contact with pathogens, the skin is a prime location for the manifestation of immune reactions. It has been proposed that the increased reactivity of EBV- or HIV-infected patients to aminopenicillines might be due to a lower activation threshold of T-cells (Posadas and Pichler, 2007). Differential pathways of activation have been show for flucloxacillin (Yaseen et al., 2015).

Severe cutaneous ADR encompass DRESS and SJS/TEN. Commonly used substances which can cause SCAR are abacavir, lamotrigine, and carbamazepine (Pirmohamed et al., 2015). The latter has an aromatic moiety and is grouped together with phenobarbital and phenytoin to the aromatic anticonvulsants. These aromatic anticonvulsants were associated with various HLA-A and HLAB variants. Interestingly, a hypersensitivity reaction to the non-aromatic drug lamotrigine was recently also associated to HLA-B*15:02 and SJS in a Han Chinese population (Man et al., 2007; Zeng et al., 2015).

The finding that abacavir is very likely to induce DRESS in HLA-B*57:01 positive patients led to a guideline of the CPIC which recommends testing because of the severity of the reaction and the high risk in absence of genetic prescreening (Martin et al., 2012). It is estimated that 6% in the general population are carriers of HLA-B*57:01 and that around 50% of carrier will develop DRESS. The recommendation is thus classified as “strong.” However, it was argued that 50% of positively tested individuals will be denied an effective treatment option (Martin et al., 2012). Therefore, CPIC reviews guidelines on a regular basis. Recently, the unchanged recommendation was confirmed (Martin et al., 2014).

**Table 2** summarizes all affected drugs and clinical manifestations of late-onset hypersensitivity reactions.

**Table 2:** Manifestations of late-onset hypersensitivity reactions and commonly affected drugs.

Affected organ	Clinical manifestation	Drugs involved
Liver	Anorexia	Flucloxacilline
	Fatigue	Amoxicilline
	Nausea	
	Abdominal pain	
	Jaundice/itching	
	Blood clotting disorders	
	Blood tests: elevated liver function tests (ALAT, ASAT)	
Granulocytes	Agranulocytosis:	Metamizole (Dipyrone)
	Sudden fever	Clozapine
	Sore throat	Carbimazol/Thiamazol/Methimazole
	Infections (urinary tract, pneumonia)	
	Sepsis	
	Blood tests: low leukocyte counts	
Skin	DRESS:	Allopurinol
	Fever	Abacavir
	Edema (face)	
	Exanthema	
	Lymphadenopathies	
	Blood tests: eosinophilia, thrombocytopenia, anemia	
	SJS/TEN:	Lamotrigine
	severe necrosis of the skin	Carbamazepine
		Phenobarbital
		Phenytoin

## Issues in Data Acquisition and Interpretation

### The Input

As nicely illustrated by the abacavir example, any improvement of signal detection and risk calculations requires correct assessment of the event (Phillips et al., 2011). Physicians in clinics tend sometimes to misdiagnose the reaction, focus on only one aspect, fail to conduct further investigations that would strengthen or invalidate the finding, or fail to document the event appropriately (Thien, 2006). The solution that worked for the abacavir-findings was to conduct two tests, both a clinical assessment using a structured query form and a skin patch test to confirm involvement of the immune system (Phillips et al., 2011).

Starting with the thalidomide tragedy, the powerful pharmacovigilance system was installed in most countries. The strength of this system is the huge data pool on adverse events like ADR encompassing millions of cases world-wide. A major drawback is the paucity of information routinely entered into this systems (Böhm et al., 2016). Genetic information is usually missing and the patients mentioned in the individual safety reports cannot be recontacted for further investigations. It is thus only possible to generate hypotheses which need further evaluation.

### The Throughput

To increase the collection of cases and their quality, interested parties have formed their own collaborative research teams, e.g., RegiSCAR for severe cutaneous reactions (Phillips et al., 2011), the Berlin Case–Control Surveillance Study group (e.g., Huber et al., 2015) and the International CIA Consortium for DIA or DILIGEN, iDILIC, DILIN, and others focusing on DILI (Nicoletti et al., 2016). These networks allow standardized collection of patient and event data as well as genetic material, and possibly re-identification of patients for further data or material sampling.

Of note, such a network requires tremendous resources if the drug-event-combination is rare. E.g., finding the association of flupirtine and HLA-DRB1*16:01-DQB1*05:02 causing DILI took more than 10 years of careful preparation and data collection (Nicoletti et al., 2016). The Berlin Case-Control Surveillance Study group collected data for 10 years (Huber et al., 2015). The increasing availability of electronic health records, the ongoing deployment of biobanks and the advances regarding the accompanying ethical, legal, technical, and social challenges (Strech et al., 2016) might deliver a global new data source for research on drug hypersensitivity reactions.

### The Output

All data essentially boils down to a 2x2 contingency table of one biomarker and the occurrence of the ADR. Several statistical measurements can be derived from this: *p*-values (e.g., derived from Chi squared with Yates’ correction), odds ratios, sensitivity/specificity, PPV, NPV as well as NNS.

It is sometimes desirable to combine several polymorphisms to a complex genotype or, analogously, several drugs to one drug class or several reactions to a syndrome. Grouping of individual findings will enhance the usefulness of the data for constructing novel detection techniques and to reach statistical and clinical significance (Böhm et al., 2016). E.g., genotyping of individual polymorphisms in cases of clozapine-induced agranulocytosis revealed no findings, whereas grouping polymorphisms to complex genotypes revealed three associations (Dettling et al., 2007). Another example is the 75% cross-reactivity of carbamazepine, phenytoine and phenobarbital and possibly lamotrigine on SJS/TEN. Arene oxide is a common moiety of metabolites of the first three anticonvulsant drugs and is considered to be the immunologic active substance. Grouping these drugs might help to strengthen signals for certain polymorphisms.

Traditional statistical approaches are not suited for analyses of small numbers. The fewer cases are reported, the wider the 95%-confidence interval becomes. The work of iDILIC shows that it is possible to find a convincing signal with just six cases (Nicoletti et al., 2016). However, in the future Bayesian approaches are expected to replace traditional frequentists’ methods for such small numbers of cases (Yum et al., 2014). Bayesian statistics is increasingly used in early clinical trials in order to efficiently screen for signals where numbers are low.

Each of the statistical measurements given above should be used for different purposes:

(i) **Statistical significance:** the *p*-value marks the feasibility for further calculations of this drug-biomarker-reaction-association.
(ii) **Technical significance:** sensitivity/specificity are useful for test validation only. They have no direct value for the patient or the health system. Of course, the higher these values are, the better the following resulting measurements.
(iii) **Clinical significance:** PPD and NPD reflect the personal risk for an individual patient. E.g., if a drug hypersensitivity reaction is extremely rare, a positive test for a biomarker does not automatically suggest a high predictive value. PPD and NPD will guide which further clinical investigations should be conducted or which treatment options are feasible.
(iv) **Economic significance:** the NNS will be used by the stakeholders of the heath system. The NNS allows to estimate the how many patients need to be screened to prevent one case of ADR. Depending on the costs of the screening procedure and the severity of the ADR, different cut-off values for NNS will be used. Time will show whether and to what extend pharmacogenetic tests will decrease the overall treatment costs for prediction of drug hypersensitivity reactions. Current evaluations estimate a plain decrease of costs for the health system (Alagoz et al., 2016).

**Table 3** shows several examples where statistical, technical, clinical and economic significance differ.

**Table 3:** Showcases of associations of drug – biomarker – event and derived statistical measurements.

#	Drug-biomarker-event	BE	Be	bE	be	*p*-value	Sensitivity/specificity	Prevalence or incidence of event	PPV/NPV	NNS	Datasource
1	Abacavir – HLA-B*57:01 – DRESS/AHSS	14	4	4	163	<0.01	77.8/97.6%	8%*	77.8/97.6%	16.58*	FD1 HLADR (Mallal et al., 2002)
2	Abacavir – HLA-B*57:01 – DRESS/AHSS	17	4	1	226	<0.01	94.4/98.3%	8%*	78.9/99.4%	15.53*	FD4 HLADR (Martin et al., 2004)
3	Abacavir – HLA-B*57:01 – DRESS/AHSS	31	8	5	389	<0.01	86.1/97.9%	8%*	79.5/98.7%	16.25*	Pooled FD1+FD4 (Mallal et al., 2002; Martin et al., 2004)
4	Abacavir – complex genotype HLA-B*57:01 + DR7 + DQ3 – DRESS/AHSS	13	0	5	167	<0.01	72/100%	8%*	100/96.9%	~12.5*	Mallal et al., 2002
5	Flupirtin – complex genotype HLA-DRB1*16:01 + DQB1*05:02 – DILI	11	0	614	10588	<0.01	1.8/100%	13.9:100,000*	100/94.5%	~8000*	Nicoletti et al., 2016
6	Flucloxacilline – HLA-*57:01 – DILI	43	8	4	60	<0.01	84.3/93.75%	8.5:100,000	0.12/99.99%	~13,000	Daly et al., 2009

AHSS, abacavir hypersensitivity syndrome; Notation of 2x2 table in adaption of (Böhm et al., 2016): BE: Biomarker + Event, Be: Biomarker but no event, bE: event but no biomarker, be: neither biomarker nor event. Values marked with * were either extracted from additional source or calculated http://openvigil.pharmacology.uni-kiel.de/contingency-table-calculator.php. FD# HLADR denotes the number of dataset obtained from http://pgx.fudan.edu.cn/hladr/.

The example of the pooled data (dataset #3) as extracted from the HLADR database shows that combining data does not substantially improve PPV, NPV, or NNS if the individual datasets are of sufficient quality (Mallal et al., 2002; Martin et al., 2004).

A common problem is the acquisition of data due to the low numbers of cases and matching controls: often, not all cells of the 2x2 contingency table contain a count. Zero counts are problematic for the calculation of measurements of disproportionality. There are several ways to cope with this situation: A mathematical tool is the usage of Haldane’s modification for Odds Ratios. By adding 0.5 to every cell, zero counts are eliminated. Another option is carefully matching other (known) cohorts to the existing data (Nicoletti et al., 2016). The theoretical gold standard is to keep gathering reports until all cells can be filled in with sufficiently large counts which is often not feasible.

The full dataset (#4) using complex genotypes illustrates that the combination of genotypes enhances the performance of the test, in this case the PPV (Mallal et al., 2002).

The dataset #5 illustrates that even test with an extremely low sensitivity can lead to acceptable high PPV and NPV and thus NNS (Nicoletti et al., 2016).

The findings in dataset #6 shows a scenario in which testing appears very reasonable if calculating PPV and NPV from the original data (PPV = 91.49%, NPV = 88.23%, NNS = 1.3). However, when applying the real population-wide prevalence instead of relying solely on the counts reported in this publication, a much lower PPD of 0.12% results and thus a very high NNS of approximately 13,000 (Daly et al., 2009). Still, testing is desirable from the patient’s point of view (NPV 100%). However, it is economically not feasible.

## When and How to Test for a Risk of Hypersensitivity

From a clinical point of view, testing for pharmacogenetics markers for the prediction of drug hypersensitivity reactions is only useful if

(i) a test with sufficient PPV/NPV exists,
(ii) an alternative treatment or diagnostic option exist that can be employed on a positive test result and
(iii) the test is unlikely to inflict damage on the patient.
From an economically point of view, it might be added:
(iv) testing of the population at risk should be cheaper than the costs to treat ADR in this population, implying a low NNS.

Of note, the NNS requires knowledge of the prevalence to be calculated. Due to the rareness of the events being analyzed, in most cases just estimates of the incidence-rates in a subpopulation (primarily users of the drug) are known. Consequently, NNS figures will vary depending on these epidemiologic data. **Table 4** lists the drugs for which currently a genetic testing prior to exposition is mandatory.

**Table 4:** Drugs for which currently pharmacogenetics testing is mandatory.

Drug	Biomarker
Abacavir	HLA- B*57:01
Carbamazepine	Only for Thai and Han Chinese: HLA-B*15:02

*Not included are any test for the existence or subsceptibility of the pharmacodynamic target (e.g., kinase inhibiting oncologic drugs and the required prior genetic testing for a mutation in the kinase).*

Sometimes, more than one test for a risk-predicting factor is available. Under most circumstances, phenotyping requires more time and could potentially damage the patient. E.g., the risk of a drug hypersensitivity reactions to abacavir can be assessed in a variety of ways: a genetic test for HLA-B*57:01, an *ex vivo* test like lymphocyte transformation test (LTT) and *in vivo* tests like a skin patch test or an oral challenge (Rive et al., 2013). The genetic test and the LTT bear no risk for the patient. The genetic test needs no cultivation of cells and is thus much cheaper than the LTT. The skin patch might induce a reaction and the oral challenge option cannot be used due to severe drug hypersensitivity reactions.

Human leukocyte antigen typing can also be done using serology, i.e., antibodies directed at certain surface proteins. However, the accuracy of this method is lower (7.1% misassignments) than genetic HLA typing (Bozon et al., 1996). Subtle changes of the HLA-encoded proteins are usually not detectable with serology-based methods. It is doubtful whether binding pockets to drugs or other interacting proteins (e.g., T cell receptors) are being recognized by the currently employed antibodies in serology-based HLA typing.

Summarizing, genetic tests have been proven to be usually the safest, fastest, and cheapest screening tool.

## Conclusion

Adverse drug reaction are a major burden for the health care system. A large percentage could be prevented. Pharmacogenetic testing can contribute to avoidance of ADR, both pharmacological ADR (type A) and drug hypersensitivity reactions (type B ADR). Certain alleles and complex genotypes of HLA genes contribute to drug hypersensitivity reactions. Common drug hypersensitivity reactions include cytotoxicity in skin, liver, and blood cells. The decision for HLA genotyping before drug therapy is dependent on severity of the expected ADR and the existence of other treatment options, as well as a reasonable high positive and NPVs of the test in question in the population to be analyzed.

## Author Contributions

IC and RB did the literature research. RB and IC wrote the manuscript.

## Conflict of Interest Statement

The authors declare that the research was conducted in the absence of any commercial or financial relationships that could be construed as a potential conflict of interest.

## Abbreviations

ACEi: angiotensin-converting-enzyme-inhibitor
ADR: adverse drug reaction
AERD: aspirin-exacerbated respiratory disease
AIU: aspirin-induced urticaria
ASA: acetylsalicylic acid
CPIC: Clinical Pharmacogenetics Implementation Consortium
CysLT: cysteinyl leukotriene
DIA: drug-induced agranulocytosis
DILI: drug-induced liver injury
DRESSs: drug rash with eosinophilia and systemic symptoms
HLA: human leukocyte antigen
LTs: leukotrienes
MCH: major histocompatibility complex
NAT: arylamine *N*-acetyltransferase
NNS: number needed to screen
NPV: negative predictive value
NSAID: non-steroidal anti-inflammatory drug
PAF: platelet-activating factor
PGs: prostaglandines
PPV: positive predictive value
SCAR: severe cutaneous ADR
SJS: Steven Johnson’s syndrome
TEN: toxic epidermal necrolysis
USAN: U.S. Adopted (Drug) Name
USAN: aspirin.
